# Diabetes mellitus and insulin resistance associate with left ventricular shape and torsion by cardiovascular magnetic resonance imaging in asymptomatic individuals from the multi-ethnic study of atherosclerosis

**DOI:** 10.1186/s12968-018-0472-9

**Published:** 2018-07-30

**Authors:** Kihei Yoneyama, Bharath A. Venkatesh, Colin O. Wu, Nathan Mewton, Ola Gjesdal, Satoru Kishi, Robyn L. McClelland, David A. Bluemke, João A. C. Lima

**Affiliations:** 1 Department of Cardiology, Johns Hopkins University, Baltimore, MD USA; 2 Offices of Biostatistics Research, National Heart, Lung, and Blood Institute, Bethesda, MD USA; 3 Department of Biostatistics, University of Washington, Seattle, WA USA; 4 National Institute of Biomedical Imaging and Bioengineering, National Institutes of Health Clinical Center, Bethesda, MD USA; 5 St. Marianna University School of Medicine, Kawasaki, Japan; 6 Radiology and Epidemiology, Johns Hopkins University, Blalock 524D1, Johns Hopkins Hospital, 600 North Wolfe Street, Baltimore, MD 21287 USA

**Keywords:** Glucose tolerance, Heart failure, Metabolic disease, Obesity, Strain

## Abstract

**Background:**

Although diabetes mellitus (DM) and insulin resistance associate with adverse cardiac events, the associations of left ventricular (LV) remodeling and function with compromised glucose metabolism have not been fully evaluated in a general population. We used cardiovascular magnetic resonance (CMR) to evaluate how CMR indices are associated with DM or insulin resistance among participants before developing cardiac events.

**Methods:**

We studied 1476 participants who were free of clinical cardiovascular disease and who underwent tagged CMR in the Multi-Ethnic Study of Atherosclerosis (MESA). LV shape and longitudinal myocardial shortening and torsion were assessed by CMR. A higher sphericity index represents a more spherical LV shape. Multivariable linear regression was used to evaluate the associations of DM or homeostasis model assessment-estimated insulin resistance (HOMA-IR) with CMR indices.

**Results:**

In multiple linear regression, longitudinal shortening was lower in impaired fasting glucose than normal fasting glucose (NFG) (0.36% lower vs. NFG, *p* < 0.05); torsion was greater in treated DM (0.24 °/cm greater vs. NFG, *p* < 0.05) after full adjustments. Among participants without DM, greater log-HOMA-IR was correlated with greater LV mass (3.92 g/index, *p* < 0.05) and LV mass-to-volume ratio (0.05 /index, *p* < 0.01), and lower sphericity index (− 1.26/index, *p* < 0.01). Greater log-HOMA IR was associated with lower longitudinal shortening (− 0.26%/index, *p* < 0.05) and circumferential shortening (− 0.30%/index, p < 0.05). Torsion was positively correlated with log-HOMA-IR until 1.5 of log-HOMA-IR (0.16 °/cm/index, *p* = 0.030).), and tended to fall once above 1.5 of log-HOMA-IR (− 0.50 °/cm/index, *p* = 0.203). The sphericity index was associated negatively with LV mass-to-volume ratio (− 0.02/%, *p* < 0.001) and torsion (− 0.03°/cm/%, *p* < 0.001).

**Conclusions:**

Glucose metabolism disorders are associated with LV concentric remodeling, less spherical shape, and reduced systolic myocardial shortening in the general population. Although torsion is higher in participants who are treated for DM and impaired insulin resistance, myocardial shortening was progressively decreased with higher HOMA-IR and torsion was increased only with less severe insulin resistance.

**Clinical trial registration:**

Multi-Ethnic Study of Atherosclerosis (MESA): A full list of participating MESA investigators and institutions can be found at http://www.mesa-nhlbi.org/. Study Start Date: January 1999 (NCT00005487).

**Electronic supplementary material:**

The online version of this article (10.1186/s12968-018-0472-9) contains supplementary material, which is available to authorized users.

## Background

Type 2 diabetes mellitus (DM) is a group of metabolic diseases; insulin resistance is a condition characterized by the failure to respond appropriately to insulin. Chronic hyperglycemia may impair myocardial energy metabolism, induces cardiomyocyte systolic dysfunction and eventually cell death [1, 2]. Epidemiological studies have demonstrated that DM and insulin resistance are related to cardiovascular disease and heart failure [3–5].

Left ventricular (LV) structure, shape and myocardial shortening (myocardial strain) have been shown to have incremental predictive value for cardiovascular events beyond the traditional LV ejection fraction measures [6–10]. Although DM and insulin resistance associate with adverse cardiac events, the associations of LV remodeling and function with compromised glucose metabolism have not been fully evaluated in a general population. We hypothesized that DM or insulin resistance might associate with LV remodeling and systolic function before developing cardiac events.

The Multi-Ethnic Study of Atherosclerosis (MESA)—a prospective study sponsored by the National Heart Lung and Blood Institute (NHLBI) of the National Institutes of Health—is a large cohort study of ethnically diverse individuals free of cardiovascular disease at baseline, and was primarily designed to study the progression of subclinical cardiovascular disease [11]. MESA is the largest cardiovascular magnetic resonance (CMR) tagging study to allow unique investigation of cardiac mechanics at the population level. To test the hypothesis, we used MESA CMR tagging examination to evaluate how CMR indices are associated with DM or insulin resistance among participants free of clinical cardiovascular disease.

## Methods

### Participants

MESA evaluated the mechanisms that underlie the development and progression of subclinical cardiovascular diseases among asymptomatic individuals. Details of the MESA study design have been previously described [11]. In brief, between July 2000 and August 2002, 6814 men and women—who identified themselves as Caucasian, African American, Hispanic, or Chinese and were 45–84 years of age and free of clinically apparent cardiovascular disease—were recruited. CMR was performed in 5004 participants as part of the baseline examination. In an ancillary study, 1773 consecutive participants underwent tagged CMR studies at enrollment in six centers: Wake Forest University, Columbia University, Johns Hopkins University, the University of Minnesota, Northwestern University, and the University of California. Of these, torsion data were available for 1478 participants as previously described [12]; of those, two cases were excluded because of lack of clinical information. A total of 1476 participants were thus enrolled in the study. None of those were known type 1 DM. All participants gave informed consent, and the study protocol was approved by the institutional review board at each site.

### Cine CMR data analysis

LV end-systolic volume and end-diastolic volume (LVEDV), LV mass, and LV ejection fraction were obtained (Additional file 1) [6, 13]. LV mass-to-volume ratio was calculated as LV mass divided by LVEDV. LV length at end-diastole was calculated as the average distance from the epicardial apex to the mitral valve insertion as measured from the 2 and 4 chamber views. The LV sphericity index at end-diastole was calculated as the percentage of the LVEDV relative to the volume of a calculated sphere with the LV length [14]. A higher index represents a more spherical shape of the ventricle and a lower reduced spericity. LV longitudinal shortening (long-axis fractional shortening) was calculated as (LV length at diastole – LV length at systole)/LV length at diastole*100 (%) [15]. A higher value represents an increased shortening of the ventricle.

Tagged CMR was performed using a segmented k-space electrocardiogram (ECG)-gated fast low angle shot pulse sequence (Additional file 1). Circumferential shortening was represented by the absolute peak strain. Positive numbers of shortening represent more contraction. Torsion (°/cm) was calculated by dividing the peak systolic twist by the inter-slice distance. CMR indices are dysplayed in Additional file 1.

### Risk factor measures

Risk factors measures are provided in Additional file 1. Body mass index (BMI) was calculated as weight over height squared. Untreated DM was defined as fasting glucose ≥126 mg/dl without any use of hypoglycemic medication or insulin. Treated DM was defined as use of hypoglycemic medication or insulin. Impaired fasting glucose (IFG) was defined as fasting glucose levels between 100 mg/dl and 125 mg/dl. All other participants were defined as having normal fasting glucose (NFG). Serum insulin was determined by a radioimmunoassay method using the Linco Human Insulin-Specific RIA kit (MilliporeSigma, Burlington, Massachusetts, USA.). Homeostasis model assessment-estimated insulin resistance (HOMA-IR) was calculated as insulin (mU/l) × (glucose [mg/dl] × 0.055)/22.5 [16, 17]. Participants with DM were excluded from the HOMA-IR calculation.

### Statistical analysis

Summary statistics were presented using median (interquartile range; 25 to 75%) for continuous variables, and percent for categorical variables. The chi-square test was used for comparison of categorical variables. Wilcoxon’s rank-sum test was used to test the differences to compare each DM fasting glucose state. Multivariable linear regression models were used to compare CMR indices by DM fasting glucose criteria. We also used multivariable linear regression to evaluate the associations of HOMA-IR with CMR indices. Potential covariates included age, gender, race, height, obesity (BMI ≥ 30 kg/m^2^), smoking status (never, former, or current), heart rate, systolic blood pressure, total cholesterol, use of medication for hypertension and dyslipidemia, estimated glomerular filtration rate, walking in METs per week, and alcoholic status (non-drinker, former, or current). Significant nonlinearity was present between the torsion and HOMA-IR with a knot at 1.5 of log-HOMA-IR. Therefore, we used a linear spline model to estimate the relationship as a piecewise linear function with adjustments for the same covariates, as described above. Standardized values were defined by dividing the differences between the observed values and the sample means by the corresponding standard deviations. Statistical analyses were performed using the Stata statistical software package (Version 14, College Station, Texas, USA). A two-sided *p*-value < 0.05 was considered statistically significant.

## Results

Of the total 1476 participants population, 210 (14%) had DM and 262 (18%) had IFG.

Baseline characteristics according to DM fasting glucose criteria are shown in Table 1. Age and systolic blood pressure were higher in the IFG group and in the Treated DM group than in the NFG group. Diastolic blood pressure was greater in the IFG group and lower in the treated DM group than in the NFG group. Hypertension was common in treated DM (NFG; 42%, IFG; 55%, untreated DM; 49%, treated DM; 74%, *p* < 0.05). Use of lipid-lowering medication was common in treated DM (NFG; 16%, IFG; 20%, untreated DM; 26%, treated DM; 33%, *p* < 0.05).

**Table 1:** Baseline demographic characteristics according to DM state (*n* = 1476)

	DM state^*^			
	NFG	IFG	Untreated DM	Treated DM
Variable	(*n* = 1004)	(*n* = 262)	(*n* = 47)	(*n* = 163)
Age, year	65 (56 to 72)	67 (61 to 72)†	66 (58 to 72)	68 (64 to 75)†
Male sex, %§	509 (50.7)	158 (60.3)	35 (74.5)	90 (55.2)
Ethnic, n (%)§				
Caucasian	336 (33)	65 (25)	12 (26)	24 (15)
Black	124 (12)	51 (19)	7 (15)	26 (16)
Hispanic	271 (27)	66 (25)	16 (34)	56 (34)
Chinese	273 (27)	80 (31)	12 (26)	57 (35)
Smoking status, n (%)				
Never	513 (51)	142 (54)	22 (47)	88 (54)
Former	376 (38)	87 (33)	20 (43)	54 (33)
Current	109 (11)	32 (12)	5 (11)	20 (12)
Alcohol status, n (%)§				
Never	195 (20)	47 (18)	8 (17)	44 (27)
Former	240 (24)	61 (23)	9 (20)	56 (35)
Current	558 (56)	152 (58)	29 (63)	61 (38)
Body mass index, kg/m^2^	27 (24 to 30)	29 (26 to 32)†	28 (25 to 32)	29 (26 to 32)†
Systolic BP, mmHg	124 (111 to 139)	131 (118 to 145)†	125 (115 to 145)	132 (120 to 146)†
Diastolic BP, mmHg	72 (65 to 78)	74 (67 to 82)†	73 (68 to 85)	70 (64 to 77)‡
Resting heart rate, bpm	60 (55 to 67)	64 (58 to 71)†	66 (60 to 71)†	66 (58 to 73)†
Hypertension, n (%)§	417 (42)	145 (55)	23 (49)	120 (74)
Current smoker, n (%)	109 (11)	32 (12)	5 (11)	20 (12)
Total-cholesterol, mg/dl	193 (172 to 215)	195 (170 to 220)	200 (170 to 218)	184 (161 to 205)
HDL-cholesterol, mg/dl	50 (42 to 60)	45 (38 to 53)†	42 (33 to 54)†	44 (38 to 54)†
Triglyceride, mg/dl	104 (74 to 150)	125 (86 to 186)†	128 (79 to 233)	128 (83 to 202)†
Lipid-lowering medication, n (%)§	160 (16)	53 (20)	12 (26)	53 (33)
Anti-hypertensive medication, n (%)§	337 (34)	128 (49)	16 (35)	122 (75)
eGFR, mL/min	76 (66 to 87)	76 (64 to 88)	83 (66 to 97)	77 (63 to 91)
Walking, MET-min/week	945 (360 to 1920)	788 (315 to 1770)	1245 (315 to 1995)	2243 (735 to 3750)

Values are median (interquartile range). BP, blood pressure; DM, diabetes mellitus; eGFR, estimated glomerular filtration rate

HDL, high-density lipoprotein; IFG, impaired fasting glucose; NFG, normal fasting glucose

^*^DM was defined as fasting glucose ≥126 mg/dl or use of hypoglycemic medication or insulin; IFG was defined as fasting glucose levels between 100 mg/dl and 125 mg/dl; all other participants were defined as having NFG

†*p* < 0.0125 vs. NFG in in Wilcoxon test, ‡p < 0.0125 vs. untreated DM in Wilcoxon test. §p < 0.05 in chi-square

### Differences in LV structure and function in DM fasting glucose criteria compared to NFG

**Fig. 1:**
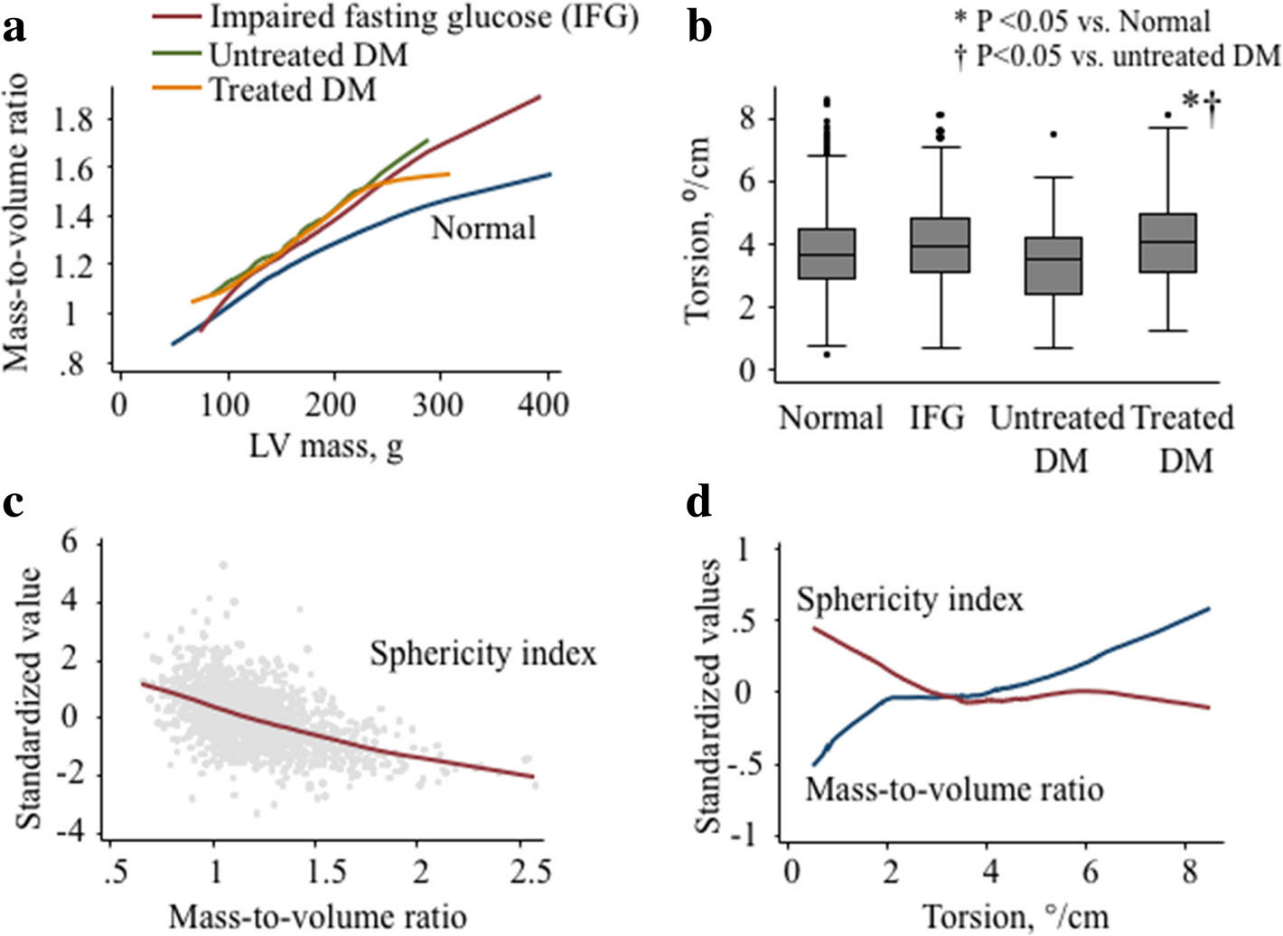
Associations of diabetes mellitus (DM) status with left ventricular (LV) indices (*n* = 1476). LV mass-to-volume ratio was higher in impaired fasting glucose and DM group than normal at any level of LV mass (**a**). Torsion was greater in treated DM than normal and untreated DM (**b**). The sphericity index was correlated negatively with the mass-to-volume ratio (**c**). Torsion was positively correlated with mass-to-volume ratio, and negatively with shericity index (**d**)

The median LV ejection fraction was 70% interquartile range (64, 74%) in the whole study population, and did not differ among DM fasting glucose criteria. Compared to NFG, treated DM tended to have greater LV mass (4.4 g greater vs. NFG, *p* = 0.077) and mass-to-volume ratio (0.04 greater vs. NFG, *p* < 0.05) with a lower longitudinal shortening (0.36% lower vs. NFG, p < 0.05) after adjustments for all prespecified confounders (Table 2). But torsion was greater in treated DM (0.24°/cm greater vs. NFG, *p* < 0.05) than NFG after full adjustments (Fig. 1). Circumferential shortening did not differ in DM status compared to NFG. The association of LV shape with concentric remodeling and torsion are shown in Fig. 1. In multiple linear regression, the sphericity index was associated negatively with LV mass-to-volume ratio (− 0.02/%, *p* < 0.001) and torsion (− 0.03°/cm/%, *p* < 0.001) after full adjustments (models included as in Table 2).

**Table 2:** Association of CMR LV indices by DM state (*n* = 1476)

Dependent variables (LV indices)	Multivariable liner regression; coefficients			
	IFG	Untreated DM	Treated DM	
	(vs. NFG)	(vs. NFG)	(vs. NFG)	R^2^
End-diastolic volume, ml	−1.86 (1.81)	2.89 (3.82)	0.17 (2.26)	0.41
Mass, g	1.64 (2.00)	5.01 (4.22)	4.42 (2.50)*	0.55
Mass-to-volume	0.04 (0.02)†	0.01 (0.04)	0.04 (0.02)*	0.19
Sphericity index, %	−0.44 (0.43)	0.94 (0.90)	− 0.27 (0.54)	0.10
Ejection fraction, %	−0.33 (0.51)	−1.27 (1.08)	0.76 (0.64)	0.19
Longitudinal shortening, %	−0.36 (0.17)†	−0.63 (0.37)*	− 0.17 (0.22)	0.17
Circumferential shortening, %	−0.03 (0.18)	−0.43 (0.39)	− 0.17 (0.23)	0.15
Torsion, °/cm	0.13 (0.08)	−0.25 (0.18)	0.24 (0.11)†	0.18

Coefficients (standard errors) represents the difference in depend variables compared to NFG as a reference in DM fasting glucose criteria. Model include age, gender, race, height, obesity (BMI ≥ 30 kg/m^2^), smoking status (non, former, and current), heart rate, systolic blood pressure, total cholesterol, use of medication for hypertension and dyslipidemia, estimated glomerular filtration rate, walking in METs per week, and alcoholic status (non-drinker, former, or current)

Abbreviations and definition of DM state as in Table 1

* *p* < 0.1, †*p* < 0.05

### LV structure and function with serum fasting glucose, insulin, and HOMA-IR

**Fig. 2:**
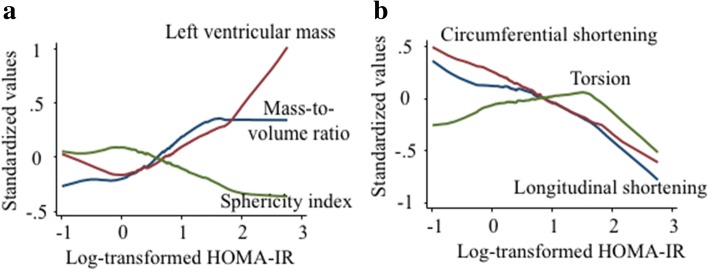
Associations of HOMA-IR with left ventricular indices among participants without diabees mellitus (*n* = 1266). Higher HOMA-IR was positively correlated with mass, and inversely with sphericity index (**a**) and myocardial shortening (**b**). The mass-to-volume ratio was flatten at the same point where the torsion reduces. Myocardial shortening was progressively decreased with higher IR. Torsion was increased only with less severe insulin resistance. Locally weighted smoothing curve (LOWESS) using unadjusted standardized values are displayed. HOMA-IR, homeostasis model assessment-estimated insulin resistance

Multivariable linear regression models in Table 3 indicate that greater log-transformed HOMA-IR was associated with greater LV mass (3.9 g/index, *p* < 0.05) and mass-to-volume ratio (0.05 /index, *p* < 0.01), and with lower sphericity index (− 1.26/index, p < 0.01) after full adjustments. Greater log-HOMA IR associated with lower longitudinal shortening (− 0.26%/index, p < 0.05) and circumferential shortening (− 0.30%/index, p < 0.05) after adjustments. These relationships were confounded by hypertension (systolic blood pressure, and use of anti-hypertensive medication) and obesity. The association between torsion and HOMA-IR was nonlinear (Fig. 2 and Table 4). Torsion was correlated positively with log-HOMA-IR until 1.5 of log-HOMA-IR (0.16 °/cm/index, *p* = 0.030), and tended to fall once above 1.5 of log-HOMA-IR (− 0.50 °/cm/index, *p* = 0.203) after full adjustments (models included as in Table 2).

**Table 3:** Association of LV remodeling and function by HOMA-IR among participants without diabetes mellitus (*n* = 1266)*

Dependent variables (LV indices)	Multivariable liner regression coefficients	
	Log-HOMA-IR	R^2^
End-diastolic volume, ml		
Model 1	2.66 (1.40)	0.36
Model 2	−2.53 (1.48)	0.41
Mass, g		
Model 1	11.58 (1.58)‡	0.49
Model 2	3.92 (1.61)†	0.56
Mass-to-volume		
Model 1	0.07 (0.01)‡	0.18
Model 2	0.05 (0.01)‡	0.20
Sphericity index, %		
Model 1	−1.06 (0.33)‡	0.11
Model 2	−1.26 (0.35)‡	0.11
Ejection fraction, %		
Model 1	0.15 (0.38)	0.18
Model 2	0.25 (0.41)	0.18
Longitudinal shortening, %		
Model 1	−0.26 (0.13)†	0.16
Model 2	−0.22 (0.14)	0.17
Circumferential shortening, %		
Model 1	−0.30 (0.14)†	0.13
Model 2	−0.08 (0.15)	0.14
Torsion, ^0^/cm		
Model 1	0.13 (0.06)†	0.16
Model 2	0.11 (0.07)	0.17

Coefficients (standard error) represents the change in dependent variables corresponds to 1 unit increase in independent variables. Model 1 included age, gender, race, height, smoking status(never, former, or current), heart rate, hypertension, total cholesterol, use of medication for dyslipidemia, estimated glomerular filtration rate, walking in METs per week, and alcohol status (non-drinker, former, or current). Model 2 included systolic blood pressure, anti-hypertensive medication use, and obesity (BMI ≥ 30 kg/m^2^) in addition to Model 1

Homeostasis model assessment-estimated insulin resistance (HOMA-IR) was calculated as insulin (mU/l) × (glucose [mg/dl] × 0.055)/22.5. [16, 17]

*Participants with diabetes mellitus were excluded because participants with DM were excluded from the HOMA-IR calculation

†p < 0.05, ‡*p* < 0.01

**Table 4:** Association of left ventricular torsion with HOMA-IR (*n* = 1266)*

	Multivariable spline liner regression		
Depend variable	Log HOMA-IR < 1.5 mg/dl	Log HOMA-IR > 1.5 mg/dl	R^2^
Torsion, °/cm			
Model 1	0.16 (0.07)†	−0.51 (.40)	0.15
Model 2	0.16 (0.08)†	−0.50 (0.40)	0.17

Coefficients (standard error) represents the change in torsion corresponds to 1 unite increase in log-HOMA-IR

Model1 included age, gender, race and height

Model 2 included age, gender, race, height, obesity (BMI ≥ 30 kg/m2), smoking status (non, former, and current), heart rate, systolic blood pressure, total cholesterol, use of medication for hypertension and dyslipidemia, estimated glomerular filtration rate, walking in METs per week, and alcoholic status (non-drinker, former, or current) as in Table 3

*Participants with diabetes mellitus were excluded as in Table 3

† *P* < 0.05

## Discussion

**Fig. 3:**
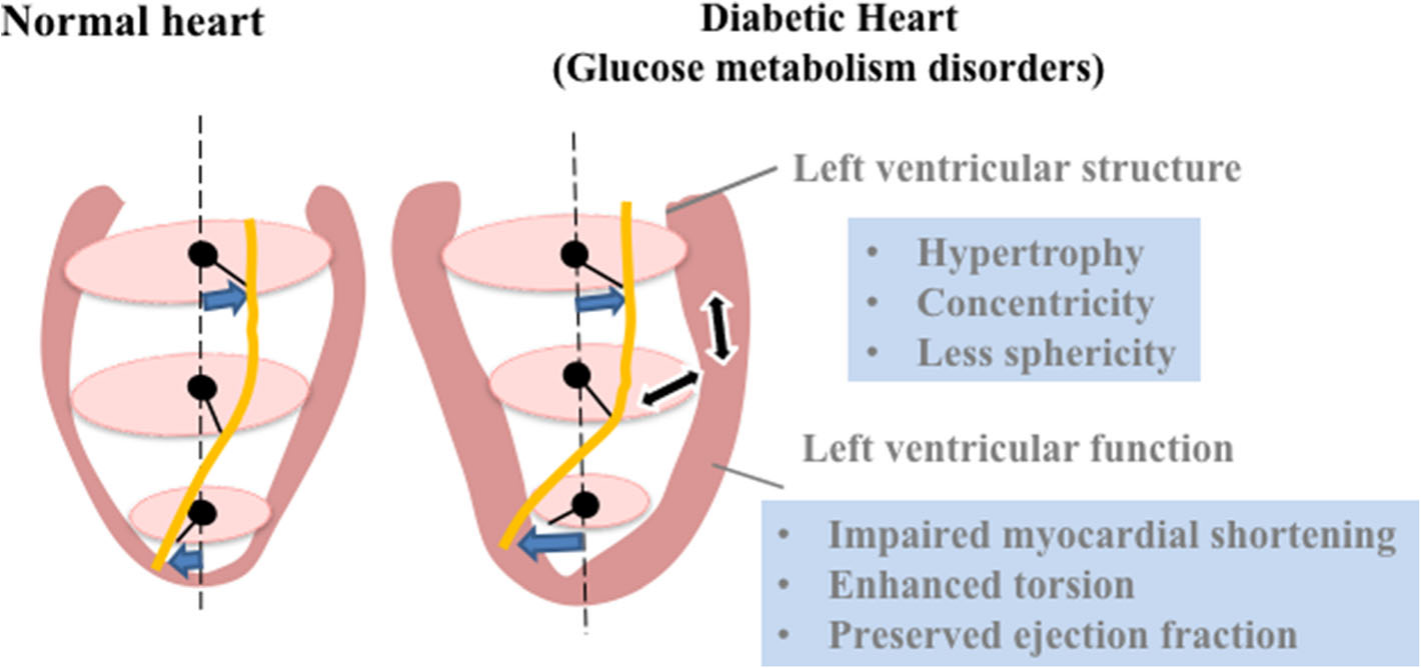
Summary of cardiovascular magnetic resonance (CMR) imaging in asymptomatic individuals. DM or impaired insulin resistance are associated with adverse LV concentric remodeling, less spherical shape, and impaired systolic myocardial shortening in the general population. Torsion, however, is higher in participants who are treated for DM and impaired insulin resistance

DM and insulin resistance are two of the most powerful risk factors for cardiovascular disease; however, the mechanisms of adaptation that alter the cardiovascular system in subjects with glucose metabolism disorders remain largely unknown. In MESA participants without known heart disease or clinical evidence of coronary artery disease at inclusion, we found the following: (1) participants with treated DM had greater LV concentric remodeling and greater torsion compared to participants with normal fasting glucose (NFG); (2) insulin resistance (non-DM) was related to a more conical LV shape and concentric remodeling with impaired systolic longitudinal myocardial shortening, but torsion was higher in advance with insulin resistance and was mirrored in a somewhat parallel manner with higher LV mass-to-volume ratio and conical shape. These observations suggest that DM or impaired insulin resistance are associated with adverse LV concentric remodeling, conical LV shape, and impaired systolic function. Increased torsion may be a key compensation mechanism to maintain global systolic function despite the alteration of myocardial shortening and structure due to compromised glucose metabolism in the general population (Fig. 3).

In the present study, subjects with treated DM had a more concentric LV than those with NFG subjects. In the Strong Heart Study, DM patients had increased LV mass and wall thickness by conventional echocardiography than normal glucose tolerance [18]. According to data from the Diabetes Control and Complication Trial/Epidemiology of Diabetes Intervention and Complications (DCC/EDIC) study, the mean Hemoglobin A1c levels are correlated positively with increased concentric remodeling defined by CMR among type 1 DM patients [19]. Our results are consistent with observations of previous large studies. We also found that higher HOMA-IR, a pre-diabetic status, was associated with concentric remodeling. The following studies also support our results: (a) in the Framingham Heart Study, LV mass and wall thickness by echocardiography were positively associated with an increase in HOMA-IR despite the relations being attenuated by BMI [20]; (b) in the Strong Heart Study, echocardiography indices demonstrated that insulin glucose tolerance was associated with higher LV mass and relative wall thickness [21]; (c) in the Coronary Artery Risk Development in Young Adults (CARDIA) study, high insulin resistance was associated with worse relative wall thickness [22]; (d) in a recent report from our study, central obesity and HOMA-IR were also associated with concentric remodeling [23].

**Fig. 4:**
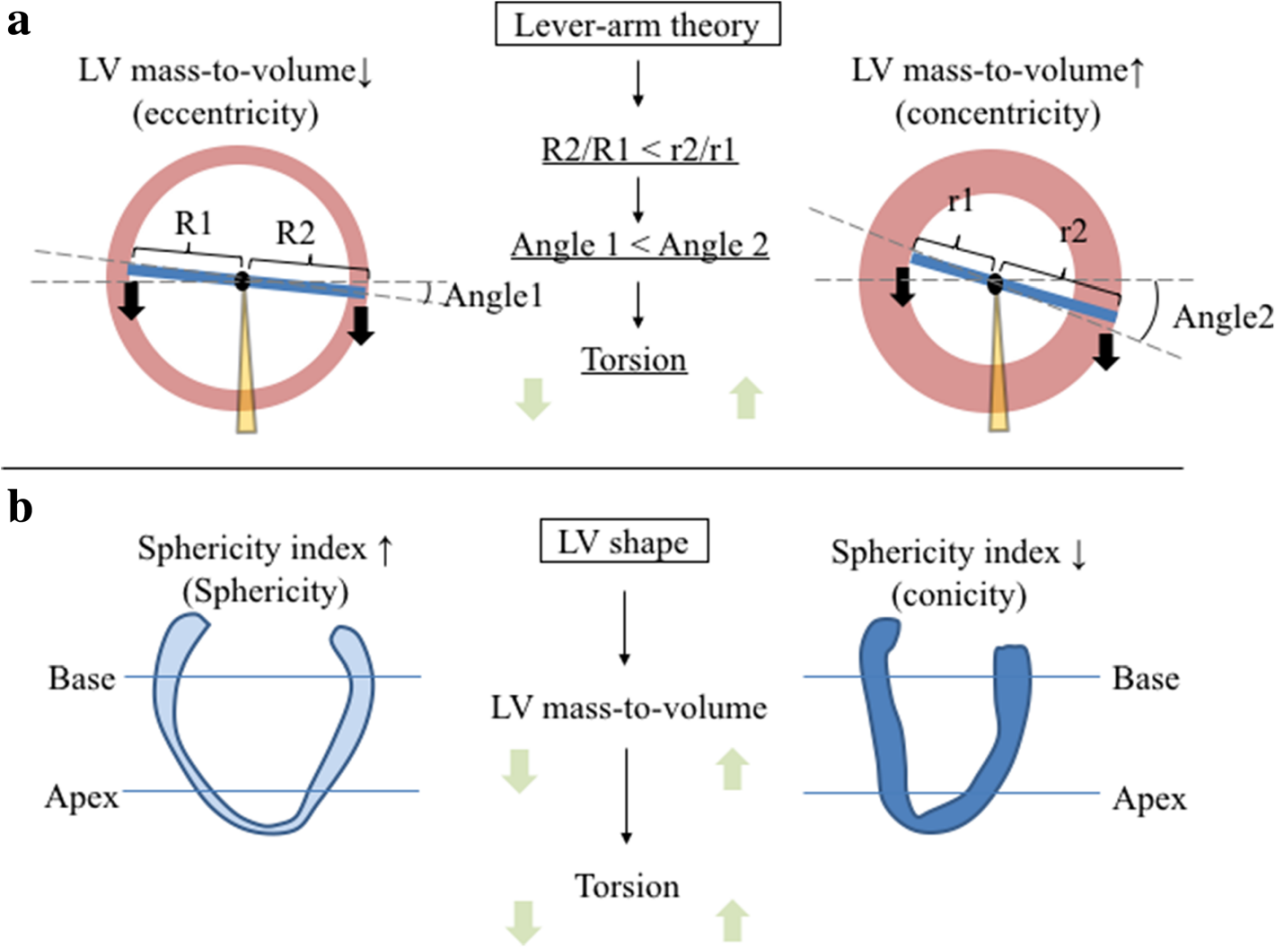
Illustrations of torsion on LV remodeling and shape geometry. **a** A greater radius difference between the endocardium and epicardium (r2/r1 > R2/R1) would result in an increased rotation angle with respect to the lever-arm theory (α1 > α2). The left arm represents the radius from center to endocardium (R1 or r1), and the right arm center to the epicardium (R2 or r2). Black arrows represent force (myocardial contraction). Dot-line represents rotation angle change. **b** Less spherical shape would result in increased LV mass-to-volume ratio, and would then influence torsion

Although the effect of LV sphericity shape has been described in LV severe systolic dysfunction [24] or in women’s hearts [12], we found that higher HOMA-IR was related to lower LV sphericity (reduced spericity). Since the more conical the LV becomes, the more the relative wall thickness increases, there might be a link between LV conical shape and torsion with respect to the lever-arm theory. In this theory, a greater radius difference between the endocardium and the epicardium would result in increased torsion (Fig. 4). Another mechanism of enhanced torsion in DM could be altered myocardial perfusion by subclinical coronary artery atherosclerosis or by coronary microvascular disease [25]. It has been reported that the apical rotation angle is internally increased by coronary occlusion in an early phase of myocardial ischemia [26], but the angle might be decreased by long-standing coronary occlusion. Shivu et al. [27] reported that enhanced torsion was associated with lower myocardial perfusion reserve assessed by adenosine stress CMR among type 1 DM patients. Since the structure of the endocardium and epicardium are oriented in different directions, reduced subendocardial function may alter the balance between the opposing rotational forces and thus result in increased torsion [28].

In the present study, torsion was not enhanced in the untreated DM group. It has been noticed in a clinical practice that the direct pathway from DM to dilated cardiac failure (increased LV sphericity and eccentricity change) can occur without a myocardial infarction [29]. Chronic exposure to disturbances of glucose metabolism in DM might negatively affect LV mechanics through myocardial fibrosis and microvascular disease [29, 30]. The spherical and eccentricity change in LV wall could attenuate enhanced torsion.

In our data, longitudinal and circumferential shortening were impaired with insulin resistance despite the relations being confounded by obesity and hypertension. In the Strong Heart Study, DM had lower fractional shortening by echocardiography than normal glucose tolerance [18]. Others have reported alterations in longitudinal and circumferential shortening by cine DENSE and 3D tag analysis in type 2 DM with small sample sizes [31, 32]. Their results agree to the current study, in that circumferential and longitudinal shortening were reduced but torsion increased, with increased mass to volume ratio. High glucose level induces an increased oxidative stress that facilitates cardiomyocyte damage and hypertrophy, and interstitial fibrosis [2]. This is in accordance with our results that reduced longitudinal and circumferential shortening are associated with increased HOMA-IR. In contrast, the data from the Framingham study of fractional shortening by M-mode did not correlate with HOMA-IR [20]. Type-1 DM with normoalbuminuria have longitudinal shortening similar to healthy control subjects [33]. Other studies have found that type-1 DM with mean hemoglobin A1c of 6.8%, had no difference in longitudinal and circumferential shortening, but torsion was increased [34]. The relatively preserved myocardial shortening in DM or insulin resistance may be partly compensated for by increased torsion. Indeed, torsion reflects the circumferential-longitudinal (CL) shear direction of the LV; therefore, the increase in torsion compensates the decrease in longitudinal and circumferential contractility.

LV torsion and myocardial strain are a principle for quantification of LV function which is also feasible with speckle-tracking echocardiography. Global LV torsion may be used to identify subclinical systolic dysfunction in patients with DM in addition to myocardial shortening. Although LV torsion has yet not been included in clinical practice guidelines, it is likely to become a useful application when evaluating patients with heart failure symptoms. Therefore, future studies should investigate if LV torsion may be useful marker of the predicting heart failure.

Our study has several limitations. First, because of the cross-sectional design, we cannot establish causal relations between glucose metabolism disorders and LV indices; however, MESA is the largest CMR tagging study to evaluate these associations so far, allowing unique investigation of cardiac mechanics at the population level. Second, the small number of untreated DM participants affects the power to detect differences in LV indices compared to NFG. This might be a factor explaining why torsion was not increased in the untreated DM group.

Although some investigators have also expressed torsion as the representing the shear deformation angle, wich torsion normalized by twist angle and LV radius [35], we did not assess the torsional shear angle.

## Conclusions

Glucose metabolism disorders are associated with LV concentric remodeling, less spherical shape, and reduced systolic myocardial shortening in the general population. Although torsion is higher in participants who are treated for DM and impaired insulin resistance, torsion was increased only with less severe insulin resistance. Increased torsion may be a key compensation mechanism to maintain global systolic function despite impaired myocardial shortening, and be explained by the changes in LV concentric remodeling with less spherical shape due to compromised glucose metabolism.

## Additional file

Additional file 1: Methods; CINE Cardiovascular magnetic resonance/ Torsion and Myocardial Strain Analysis/ Left ventricular indices/ Risk factor measures. (DOCX 636 kb)

## Abbreviations

BMI: Body mass index
CMR: Cardiovascular magnetic resonance
DM: Diabetes mellitus
ECG: Electrocardiogram
HOMA-IR: Homeostasis model assessment-estimated insulin resistance
IFG: Impaired fasting glucose
LV: Left ventricle/left ventricular
LVEDV: Left ventricluar end-diastolic volume
NFG: Normal fasting glucose
MESA: Multi-Ethnic Study of Atherosclerosis
