# Supplementary material for: Diabetes mellitus and insulin resistance associate with left ventricular shape and torsion by cardiovascular magnetic resonance imaging in asymptomatic individuals from the multi-ethnic study of atherosclerosis
Source: J Cardiovasc Magn Reson. 2018 Jul 30;20:53. doi: 10.1186/s12968-018-0472-9 (PMC6069876; doi:10.1186/s12968-018-0472-9)
Supplement: Supplementary file 1 — Methods; CINE Cardiovascular magnetic resonance/ Torsion and Myocardial Strain Analysis/ Left ventricular indices/ Risk factor measures. (DOCX 636 kb) [file 12968_2018_472_MOESM1_ESM.docx]

Additional file 1 online for

**Diabetes Mellitus and Insulin Resistance Associate with Left Ventricular Shape and Torsion by Cardiac Magnetic Resonance Imaging in Asymptomatic Individuals**

**from the Multi-Ethnic Study of Atherosclerosis**

Kihei Yoneyama, Bharath A. Venkatesh, Colin O. Wu, Nathan Mewton, Ola Gjesdal, Satoru Kishi, Robyn L. McClelland, David A. Bluemke, João A. C. Lima, MD^*^

*Corresponding author e-mail: jlima@jhmi.edu

SUPPLEMENTRAY APPENDIX

Table of Contents

### Additional file 1 (CINE Cardiac magnetic resonance)…………………..…Page 3

- **Additional file 2 (Torsion and Myocardial Strain Analysis)…………….…Page 4**
- **Additional file 3 (Figure)…………….……………………………….………Page 6**
- **Additional file 4 (Risk factor measures)…………….……………….………Page 7**

**Methods**

### CINE Cardiac magnetic resonance (CMR)

CMR examinations were performed at 6 centers (Baltimore, Winston-Salem, New York, Minneapolis, Los Angeles, and Chicago) using either a Signa Excite (General Electric Medical Systems, Waukesha, Wisconsin) or Avanto/Espree (Siemens, Erlangen, Germany) 1.5-T MR scanners for examinations baseline and follow-up. Planning of the cardiac cine images for both examinations was standardized to minimize variation among centers. Cine images were obtained with a temporal resolution of approximately 50 ms or less using a segmented k-space and an electrocardiographically gated, fast spoiled gradient-recalled echo pulse sequence. To determine the LV mass, the difference between the epicardial and endocardial areas for all slices was multiplied by the slice thickness and section gap, and then multiplied by the specific gravity of the myocardium (1.04 g/ml). Left ventricular end-systolic volume and end-diastolic volume, left ventricular mass, and left ventricular ejection fraction were measured using commercially available software (MASS 4.2, MEDIS+, Leiden, The Netherlands). At follow-up, participants without contraindications underwent CMR exams using 1.5T scanners (Avanto and Espree, Siemens Medical Systems; Signa LX, GE Healthcare) with a six‐channel anterior phased‐array torso coil and corresponding posterior coil elements. Left ventricular function, dimensions and myocardial mass were assessed by a cine steady‐ state free precession sequence. Left ventricular volumes and mass were assessed by a nine steady state-free precession sequence using commercially available software (CIM v6.2, Auckland, New Zealand).

**Methods**

**Torsion and Myocardial Strain Analysis**

Both baseline and follow-up tagged CMR were performed using a segmented k-space ECG-gated fast low angle shot pulse sequence. Dedicated phase array coils were used for signal acquisition. After concluding the standard imaging protocol, three tagged short-axis slices were acquired at the LV base, midlevel, and apex. Parallel striped tags were prescribed in two orthogonal orientations (0° and 90°) using identical pulse sequence with additional spatial modulation of magnetization. The parameters for tagged CMR images were: field of view 40 cm; slice thickness 8 to 10 mm; repetition time 3.5 to 7.2 ms; echo time 2.0 to 4.2 ms; flip angle 12°; matrix size 256 × 96 to 140; 4 to 9 phase-encoding views per segment; temporal resolution 20 to 41 ms; and tag spacing 7 mm. Three tagged short-axis slices were obtained (base to apex) with 2 orthogonally oriented parallel striped tags (0° and 90°) using spatial modulation of magnetization,

Rotation and circumferential shortening were assessed in short axis tagged slices using HARP software (Harmonic Phase, Diagnosoft, Palo Alto, CA). After images importing and superimposing the 2D stripe tags in vertical and horizontal direction, endocardial and epicardial contours were manually traced on the image corresponding to the remaining cardiac phases within a few seconds automatically. A few interactive corrections of the contour tracking were performed when necessary for obtaining satisfactory matching. The anterior attachment of the right ventricular wall to the left ventricular was always chosen as the landmark of reference for clockwise numbering of the segments. And then HARP computed the rotation and strains for single cardiac phase. Rotation (°) was defined for each short axis slice as average angular displacement in the LV midwall layer. During a normal systole, the basal rotates in a clockwise, and the apex in a counterclockwise direction when viewed from the left ventricular apex. Normal apical rotation is by definition positive; twist (°) was calculated as the net difference between apical and basal rotation angle for each frame during the cardiac cycle using MATLAB ® (The MathWorks, Natick, MA, USA). Because an estimation of the peak twist calculated as the difference between peak rotation in between the basal and apical slices may overestimate peak LV twist if the timing of peak rotations are different between the slices. To normalize twist for slice distance, torsion (°/cm) was calculated by dividing peak systolic twist by the inter-slice distance h (cm). The distance h was calculated as the sum of one image plane thickness and the gap between planes. Strains express the fractional change in length (as percentage) from a resting state (end-diastole) to one achieved following myocardial contraction. Strain was defined by the formula; strain = ( *L*_d_ - *L*_r_) / *L*_r_. Where *L*_d_ is the length in the deformed state and *L*_r_ is the length in the relaxed state (end diastole). Circumferential shortening was represented by the absolute peak strain value and determined and averaged from 4 LV segments (anterior, lateral, inferior, and septal) from the LV midwall layer on the mid-ventricular slice. Positive numbers represent more shortening.

**Figure 1. Left ventricular indices by cardiac magnetic resonance imaging**

LV sphericity index (a), and longitudinal shortening (b) was obtained from Cine. Torsion was obtained from tagged cardiac magnetic resonance (c).

**Methods**

**Risk factor measures**

Standardized questionnaires were used to obtain information about smoking history and medication usage, and for history of high blood pressure and diabetes. Smoking was defined as never, former (smoked ≥100 cigarettes in lifetime), or current (smoked cigarettes in last 30 days). Participants’ height and weight were measured, and body mass index was calculated as mass in kilograms divided by height in meters squared. Resting blood pressure was measured three times with participants in a seated position using a Dinamap model Pro 100 automated oscillometric sphygmomanometer. The average of the last 2 measurements was used. Hypertension was defined as systolic blood pressure ≥140 mm Hg or diastolic blood pressure ≥90 mm Hg, or self-reported hypertension and antihypertensive medication use.^1^ High-density lipoprotein cholesterol (HDL-C) and other laboratory assays were measured from blood samples obtained after a 12-h fast. Lipid, lipoprotein, and other laboratory assays. Blood was drawn after a 12-h fast, and samples were stored at −70°C. Lipids, and glucose were measured at a central laboratory (Collaborative Studies Clinical Laboratory at Fairview University Medical Center, Minneapolis, Minnesota). Lipids were assayed on thawed ethylenediaminetetraacetic acid plasma within 2 weeks of the sample collection, using Centers for Disease Control Prevention/NHLBI standards. HDL-C was measured using the cholesterol oxidase method (Roche Diagnostics, Indianapolis, Indiana) after precipitation of non–HDL-C with magnesium/dextran (coefficient of variation 2.9%). The glomerular filtration rate was estimated with the MDRD / CKD EPI/ Cockroft-Gault equation.^2^

**References**

1. Chobanian AV, Bakris GL, Black HR, Cushman WC, Green LA, Izzo JL, Jr., Jones DW, Materson BJ, Oparil S, Wright JT, Jr., Roccella EJ. The Seventh Report of the Joint National Committee on Prevention, Detection, Evaluation, and Treatment of High Blood Pressure: the JNC 7 report. *JAMA* 2003;**289**(19):2560-72.

2. Matsushita K, Mahmoodi BK, Woodward M, Emberson JR, Jafar TH, Jee SH, Polkinghorne KR, Shankar A, Smith DH, Tonelli M, Warnock DG, Wen CP, Coresh J, Gansevoort RT, Hemmelgarn BR, Levey AS, Chronic Kidney Disease Prognosis C. Comparison of risk prediction using the CKD-EPI equation and the MDRD study equation for estimated glomerular filtration rate. *JAMA* 2012;**307**(18):1941-51.
